# Fast Adapting Ensemble: A New Algorithm for Mining Data Streams with Concept Drift

**DOI:** 10.1155/2015/235810

**Published:** 2015-03-23

**Authors:** Agustín Ortíz Díaz, José del Campo-Ávila, Gonzalo Ramos-Jiménez, Isvani Frías Blanco, Yailé Caballero Mota, Antonio Mustelier Hechavarría, Rafael Morales-Bueno

**Affiliations:** ^1^Department of Computer Science, University of Granma, 85100 Granma, Cuba; ^2^Department of Language and Computer Science, University of Málaga, Complejo Tecnológico, 29071 Málaga, Spain; ^3^Department of Computer Science, University of Camagüey, 70100 Camagüey, Cuba

## Abstract

The treatment of large data streams in the presence of concept drifts is one of the main challenges in the field of data mining, particularly when the algorithms have to deal with concepts that disappear and then reappear. This paper presents a new algorithm, called Fast Adapting Ensemble (FAE), which adapts very quickly to both abrupt and gradual concept drifts, and has been specifically designed to deal with recurring concepts. FAE processes the learning examples in blocks of the same size, but it does not have to wait for the batch to be complete in order to adapt its base classification mechanism. FAE incorporates a drift detector to improve the handling of abrupt concept drifts and stores a set of inactive classifiers that represent old concepts, which are activated very quickly when these concepts reappear. We compare our new algorithm with various well-known learning algorithms, taking into account, common benchmark datasets. The experiments show promising results from the proposed algorithm (regarding accuracy and runtime), handling different types of concept drifts.

## 1. Introduction

Classification algorithms that learn from data streams in the presence of concept drifts have received a lot of attention in recent years. They are very important because of their application in different areas such as bioinformatics, medicine, economics and finance, industry, the environment, and many other fields of application. For instance, Gama et al. [1] have grouped the applications requiring adaptation into four categories: monitoring and control, management and strategic planning, personal assistance and information, and ubiquitous environment applications.

Within incremental learning [2], the problem of classification is generally defined for a sequence (possibly infinite) of examples (also known as instances) *S* = *e*_1_, *e*_2_, …, *e*_*i*_,… arriving over time, normally one at a time and not necessarily time-dependent. Each training example $e_{i}=(\vec{x_{i}},y_{i})$ is formed by a vector $\vec{x_{i}}$ and a discrete value *y*_i_, named label which is taken from a finite set *Y* named class. Each vector $\vec{x_{i}}\in \vec{X}$ has the same dimensions, each dimension is named attribute and each component *x*_*i*,*j*_ is an attribute value (numeric or symbolic). It is assumed that there is an underlying function $y=f(\vec{x_{i}})$ and the goal is to obtain a model from *S* that approximates *f* as $\hat{f}$ in order to classify or predict the label of nonlabeled examples (also known as observations), so that $\hat{f}$ maximizes the prediction accuracy [3]. Sometimes it is assumed that the examples arrive in batches of the same size. Let us consider concept as the term that refers to the whole distribution of the problem at a certain point in time [4]. This concept can be characterized by the joint distribution $P(\vec{X},Y)$.

In the real world, concepts are often unstable and change over time. The underlying data distribution may change as well. Often these changes make the model built on old data inconsistent with the new data and an updating of the model is necessary. This problem, known as concept drift, complicates the task of learning a model from data and requires an additional mechanism in order to maintain the learning model up-to-date with respect to the current concept [5].

According to Tsymbal [5], an ideal concept drift handling system should be able to (1) quickly adapt to concept drift, both abrupt and gradual; (2) be robust to noise and be able to distinguish it from concept drift; and (3) recognize and treat recurring contexts. However, often the mechanisms, used to favor a fast adaptation to concept drifts, like, for example, the use of base classifiers that individually adapt to that change and to their rapid substitution with current classifiers, make the correct treatment of the recurring concepts more difficult.

On the other hand, today's ensemble systems have gained in importance as they provide a mechanism that effectively combines a set of classifiers to obtain not only a more complex but also a more accurate classification model [6].

In this paper, we present Fast Adapting Ensemble (FAE), an algorithm that adapts very quickly to both abrupt and gradual concept drifts and has been specifically built to deal with recurring concepts.

## 2. Related Work

Gama et al. [7] distinguish two categories in which strategies are positioned to address the problem of concept drift: strategies in which learning adapts at regular time intervals without considering that there has been a change in the concept and strategies in which a concept drift is first detected, and then learning adapts to this change. Ensembles are usually included within the first strategy, as they have mechanisms (to update existing classifiers, to eliminate low-performance classifiers, to insert classifiers, etc.) that allow them to evolve without having to directly detect concept drift. However, recent research proposes different mechanisms of direct detection of changes that are inserted into the ensembles. One advantage of incorporating a drift detector is to exploit the capacity of ensembles to adapt to gradual changes, combined with the natural working mode of the detector during abrupt changes.

### 2.1. Ensembles for Data Stream Mining

One of the first proposals for data stream mining was the Streaming Ensemble Algorithm (SEA) [8]. SEA divides the training dataset into batches of the same size and a new base classifier is built from each one of these batches and added to the ensemble. The algorithm has a maximum number of classifiers that, when reached as an adaptation mechanism, requires the replacement of previous base classifiers by following certain criteria. To unify the predictions of the base classifiers, SEA uses unweighted-majority voting. SEA adapts to gradual changes well, but its adaptation is not as good for abrupt changes. According to Kolter and Maloof [9], these results are influenced by the voting mechanism used and also because classifiers stop learning once they have been created. An algorithm which follows a similar scheme to SEA is MultiCIDIM-DS, proposed by del Campo-Ávila [6].

Under the same division scheme of the training dataset, Wang et al. [10] proposed a new method, called Accuracy Weighted Ensemble (AWE). To combine the response of base classifiers, the proposal uses a weighted-majority voting. The weighting of the base classifiers depends on the accuracy obtained by them when using the examples from the current training batch. As SEA, it adapts to gradual changes, but it has trouble adapting to abrupt concept drifts. One of the reasons for this inefficiency is that AWE has to wait for the next batch in order to update the weights of base classifiers. Unfortunately, reducing the size of the batch does not solve the problem because that would result in lower overall system accuracy.

The Batch Weighted Ensemble algorithm (BWE) [11] is an ensemble that takes the AWE algorithm as its basic precursor. This proposal is one of those included within the second strategy proposed by Gama et al. [7]; therefore, it incorporates a drift detector inside the model; this detector is called the Batch Drift Detection Method (BDDM) and uses a regression model to determine the presence of concept drift. The drift detector is basically used to determine whether to create a new base classifier due to concept drifts, or whether the concept is stable and the ensemble has not been modified. The idea is to combine the ability of the ensembles to adapt to gradual changes with the natural working mode of the drift detector for detecting abrupt changes.

According to Gonçalves and Barros [12], the Accuracy Updated Ensemble (AUE) [13] is an enhancement of AWE. Both use classifier ensembles and are associated with weights that are updated as data arrive. The main difference between them is the usage of incremental classifiers instead of static ones; it proposes a simpler weighting function to avoid zeroing the weight of all classifiers, a possible situation in AWE, and updates classifiers only if they have been highly accurate in recent data.

Another idea for data stream mining is to use the training examples one by one as they arrive, online. An algorithm that uses this system to update its base classifiers is the Dynamic Weighted Majority (DWM), proposed by Kolter and Maloof [9]. DWM is based on the Weighted Majority Algorithm (WMA) [14], which takes the idea of working with a group of experts, to which an initial weight is automatically assigned. Then, when a new example arrives, the base algorithm receives a prediction from each expert and makes a final decision by combining the predictions and the weights of each expert; finally, if an expert makes an incorrect prediction, then its weight is reduced by a multiplicative constant between 0 and 1. In order to adapt to working with data streams and to handle concept drifts, DWM includes mechanisms to add, update, and delete base classifiers. At each given moment *p*, a test is performed and a new classifier is added with a weight value equal to 1 if the system output is incorrect; moreover, the system deletes each base classifier, whose weight falls below a threshold of *θ*. One of the potential problems of this algorithm is that it penalizes base classifiers when they fail but it does not reward them when they are right; this makes the base classifiers' weights fall quickly and they only remain a short while within the ensemble; this, coupled with the fact that DWM steadily updates the base classifiers, does not make it suitable for the treatment of recurring concepts.

Kolter and Maloof also proposed an algorithm called the Additive Expert Ensemble (AddExp) [15]. This system is very similar to DWM and both have common mechanisms such as the type of voting, the way of inserting new classifiers, and the mechanism, to quickly remove multiple classifiers simultaneously. They differ in the fact that they propose two distinct methods for replacing the classifiers: the first one is based on removing the old ones, for which a constant that controls how long the expert has been within the ensemble is included, and the second one is based on the weakest classifier, as it deletes the one with the lowest weight. Similar to DWM, AddExp inherits the same deficiencies in the treatment of recurring concepts.

With the same work strategy with the data stream, the Ensemble Classification Algorithm for Incremental Data Streams (ICEA) was proposed [16]. The idea of this proposal is that each base classifier learns incrementally, automatically adding the result of their learning as quickly as possible. According to the authors, a faster detection of the concept drift is obtained, when compared to some batch-based algorithms. ICEA uses adapting mechanisms similar to those of DWM. As in DWM, classifiers may be only a short time within the ensemble, which makes it inefficient to handle recurring concepts, this in addition to the fact that base classifiers are steadily readapted, forgetting the old concepts.

The DWM-WIN algorithm [17] proposed some modifications to the DWM algorithm. The first modification is based on a characteristic of the version of the Winnow algorithm implemented by Blum [18]. Winnow is similar to WMA in the idea of changing the weight of the experts according to their individual prediction; the difference is that it includes a new multiplicative constant (*η* > 1) to reward the expert weight when the prediction is correct. By adding this feature, DWM-WIN ensures that each expert is more likely to stay within the ensemble if their behavior improves over time; this makes it more flexible when dealing with recurring concepts. Another modification is that, in some variants of the proposed algorithm, when removing experts, their age is taken into account.

A new ensemble for incremental learning, named Diversity for Dealing with Drifts (DDD), is proposed by Minku and Yao [19]. DDD maintains several ensembles with different levels of diversity. If the presence of concept drifts is not detected in the data, the system will consist of two ensembles, one with a low diversity and one with a high diversity. When a concept drift is detected, two new ensembles are built, one with a low diversity and one with a high diversity. According to the authors, old ensembles are maintained because this ensures a better exploitation of diversity, the use of the information learned from old concepts and robustness against false alarms. The four ensembles are maintained while two conditions that check the change status are met; otherwise, using a combination mechanism, a working model with two ensembles starts again. The authors report that DDD is able to maintain a better accuracy than other proposals such as DWM.

Finally, there has been a recent addition to proposals that adapt the well-known algorithms Bagging [20] and Boosting [21] for data stream mining. Using a heuristic and a weighted majority voting, Bagging and Boosting are algorithms which create intermediate models that are the basis for a single final model whose accuracy improves the accuracy of any one of them. According to the Bagging algorithm, the final model is made from the most common rules within several individual models, and according to the Boosting algorithm, multiple classifiers, which are voted according to their error rate, are generated, but unlike the Bagging algorithm, they are not obtained from different samples but rather sequentially on the same training set. Incremental versions of the Bagging and Boosting algorithms have been proposed by Oza and Russell since 2001 [22]. But, other versions that adapt to concept drifts have appeared more recently.

The OzaBagADWIN algorithm proposed by Bifet et al. [23] is a Bagging algorithm adaptation. The idea of this proposal is to add a drift detector called Adaptive Windowing (ADWIN) [24] to the incremental version of the Bagging algorithm [22]. The adaptation mechanism is based on replacing the worst of the classifiers in an instant of time with a new base classifier created more recently.

The Adaptive Boosting Ensemble Classifier (ACS) [25] is an adaptive version of Boosting algorithm proposed by Wankhade and Dongre. This new version uses the Boosting algorithm for an ensemble method combined with an adaptive sliding window and a Hoeffding tree to detect concept drifts, and if necessary, add a new base classifier; this mechanism improves the functioning of the ensemble. According to the author, the algorithm works well in environments with concept drifts, as it adapts dynamically and quickly to changes and it also requires little memory to operate.

Another adaptive version of the Boosting algorithm was proposed by Dongre and Malik [26]. The new adaptation follows a similar idea to ACS but combines the well-known Boosting algorithm with the ADWIN drift detector [24]. The proposal uses the Boosting algorithm as the ensemble method and ADWIN to detect concept drifts and if necessary handle the input data window and add new base classifiers. The results show that the proposed method takes less time, uses less memory, and is more accurate than other known methods (OzaBag, OzaBoost, and OzaBagADWIN).

None of the aforementioned classifiers take into account the possible presence of recurring concepts, so they have not been adapted to work with them.

### 2.2. Systems for the Treatment of Recurring Concepts

The online learning system should be able to recognize and handle recurring concepts. If a concept has appeared before, previous successful classifiers should be used. Using many classifiers built from old concepts is one possible way to handle recurring concepts.

The Adaptive Classifiers Ensemble (ACE) [27] is a system, published by Nishida et al., which is able to handle recurring concepts better than a conventional system. This ensemble is accompanied by four elements: first, a single classifier that uses the input data one by one incrementally; this classifier replaces the ensemble for the prediction work when abrupt concept drifts take place because the ensemble takes a long time to update as it has to wait for the next batch to arrive to do so; second, a drift detector; third, a sliding window used to store the results of predictive accuracy and confidence intervals of each classifier on the most recent data, and finally, a buffer used to store recent training examples and to build the new classifiers.

An ensemble especially for the treatment of recurring concepts was presented by Ramamurthy and Bhatnagar [28]. This approach builds a historical global set of classifiers (decision trees) from sequential data chunks of same size. Each individual classifier for this committee represents a different concept. So a new classifier is only built when the concept in the data stream changes and when this concept is not represented by a classifier in the historical global set. These historic classifiers are never deleted because the concept that one represents may reappear. Not all the classifiers participate in the classification process at the same time. The system uses a filter which screens the existing classifiers and allows only those relevant to the current concept to participate in the classification process. This approach, like AWE, has to wait for the next chunk in order to update all the mechanisms of the system.

Although not an ensemble, the algorithm, Recurring Concept Drifts (RCD) [12], is included here because it is able to handle recurring concepts. RCD is not a simple classifier nor an ensemble, but rather a collection of classifiers from which the one to be used is selected at any time based on the distribution of the current data; for this, nonparametric statistical tests are used. A new classifier and a significant sample of the data used to create it are added to the collection each time a new detected concept fails to match any of the previously stored concepts. The authors state that their results are superior to those of other algorithms when faced with abrupt changes and they get similar results when addressing gradual changes.

Finally, we have included two other approaches that are not ensembles but are able handle recurring concepts. Li et al. [29] proposed a classification algorithm called REDLLA for data streams with recurring concept drifts and limited labeled data. It was built for semisupervised learning and it adopts a decision tree as the classification model. When growing a tree, a clustering algorithm based on k-means is installed to produce concept clusters and to label unlabeled data at leaves. In the presence of deviations between historical concept clusters and new ones, potential concept drifts are distinguished and recurring concepts are maintained. According to the authors, REDLLA algorithm is efficient and effective for mining recurring concept drifts even in cases with a large volume of unlabeled data.

Gama and Kosina [30] present a method that memorizes learnt decision models whenever a concept drift is signaled. The system uses meta-learning techniques that characterize the domain of applicability of previous learnt models. The meta-learner can detect the reoccurrence of contexts and take proactive action by activating previously learnt models. According to the authors, the main benefit of this approach is that the proposed meta-learner is capable of selecting similar historical concepts, if indeed such exist, without the knowledge of true classes of examples.

## 3. Fast Adapting Ensemble: A New Ensemble Method

As shown in the previous section, there are a few proposals that use ensembles to treat recurring concepts. The use of base classifiers which individually adapt to change and the little time they sometimes remain within the ensemble favor a fast adaptation to concept drifts but make the correct treatment of recurring concepts more difficult.

FAE is an ensemble designed to quickly adapt to concept drifts and specializes in the treatment of recurring concepts. Like RCD [12], this proposal has a set of classifiers that represents several of the concepts analyzed; although it differs in that, these classifiers are organized into active and inactive, according to their behavior when testing current data. FAE is an ensemble that takes its global decision from the partial decision of the active classifiers, while retaining a group of inactive classifiers as a warehouse of old concepts, which ease the treatment of recurring concepts. These inactive classifiers are activated very quickly if the concept that they represent reappears. Reactivation of classifiers and insertion of new updated classifiers, if necessary, ensure rapid adaptation, especially if the concepts are recurring.

Like several of the algorithms analyzed [6, 8, 10, 11], FAE divides the training data stream into blocks of the same size and builds, if necessary, a new base classifier, which adds to the ensemble; thus naturally, it obtains knowledge from large datasets. The algorithm sets a maximum limit of classifiers to store, which, when reached as an adaptation mechanism, requires replacing previous classifiers following certain base criteria.

FAE associates a weight to each base classifier and uses weighted-majority voting to unify the partial votes. Like Wang et al. [10], in order to update the weights, it uses the precision obtained by each of the base classifiers when testing the current training set but differs in that FAE proposes a new formula for adjusting the weights and it also does not have to wait for the new training block to be completed but continues updating the weights of the base classifier with parts of the block. Due to the characteristics of the formula for updating, the weight associated with each base classifier may decrease or increase depending on its behavior when testing the new data.

This proposal is included in the second strategy mentioned by Gama et al. [7] because it incorporates a drift detector to the model. As discussed by Deckert [11], the drift detector is used to determine when to create a new base classifier according to the presence or absence of concept drifts; if the concept is stable, an unnecessary new classifier is not created, which contributes to saving memory and favors previous base classifiers representing other concepts remaining within the ensemble. The purpose of this idea is to take advantage of the capacity of ensembles to adapt to gradual changes combined with the natural work of the drift detector for abrupt changes. Because of this, FAE is able to manipulate both gradual and abrupt concept drifts (see Algorithm 1).

### 3.1. Initialization of the Ensemble

For the ensemble to be functional, though, of course, not properly trained, it is necessary to ensure that there is at least one active base classifier. For this reason, the initial step is to create, with the first block of training, a base classifier with its active status and initial weight equal to 1. In addition, the first concept to be analyzed is initialized (see Algorithm 2).

### 3.2. Update the Weights and Status of the Base Classifiers

The weights and status of base classifiers are updated each nt period. The value nt is the number of examples needed to update the weights and status of base classifiers; this value should be less than that defined for a set of examples (ne number of examples needed to create a new base classifier), in order not to wait unnecessarily for a block to be completed to update the base classifier weight. This is one of the shortcomings of the algorithm proposed by Wang et al. [10], which is why it was difficult to detect abrupt concept drifts.

The formula used to update the weights is inspired by studies in disciplines such as telecommunications [31], specifically formulas for smoothing to calculate a stable measure of the usability of communication lines. This way of updating the weights of the classifiers allows them to be increased or decreased according to the behavior of the classifiers when testing the current training set. It is intended that the base classifiers can remain longer within the ensemble.

Preset constants *β*_1_ and *β*_2_ (*β*_1_ + *β*_2_ = 1) represent the level of importance they have given to the behavior of base classifiers over old data and current data, respectively. A high value of *β*_1_ (compared to the value of *β*_2_) means that more importance will be given to the historical behavior of the classifier than to its behavior over current data; change adaptation will be a little slower but the process will be more robust over noisy data. A high value of *β*_2_ (compared to the value of *β*_1_) means that more importance will be given to the current behavior of base classifier than to its historical behavior; change adaptation will be much faster but it is likely to be affected by noisy data. Hence, the importance of assigning values to *β*_1_ and *β*_2_ is directly related to the balance desired between sensibility to concept drifts or noisy data (see Algorithm 3).

It is important to note the fact that inactive classifier weights are not decreased; they are only increased if current classifier behavior improves. The purpose of this procedure is not to unnecessarily reduce the weight of a classifier, about which it is known that it has not been identified with the current concept (for this reason it is inactive), and thus rapid activation is ensured when the concept that it represents appears again.

The base classifier can have two statuses, active or inactive. An active classifier is one that keeps its weight above a preset threshold *θ*. For predicting in an instant of time, only active classifiers are used, as they are considered the best adapted to the current concept.

An inactive classifier is one that keeps its weight below the preset threshold *θ*. Inactive classifiers are not involved in predictions but remain stored as long as possible, as they represent old concepts. The weights of inactive classifiers are also updated every nt examples (only if it is improved) (see Algorithm 4).

Whenever weights of base classifiers are updated; afterwards, statuses are updated, too; thus the activation-inactivation of the classifiers in the ensemble is ensured.

There are two implementation details not reflected in the pseudocode: first, when updating the statuses of base classifiers, it is always ensured that at least one classifier remains in the active status and also that it has the best current behavior (highest weight); second, in the experiments, a somewhat higher value than the threshold *θ* is used to activate a base classifier; with it, subsequent changes of activation-inactivation or vice versa, which are annoying and harmful to predictions, decrease.

### 3.3. Adding a New Base Classifier

The first thing to be considered in order to add a new base classifier is the information provided by the drift detector used. The drift detector must have as output three possible alerts: no change, possible change (warning), and change (drift). The known drift detectors DDM (drift detection method) [32] proposed by Gama et al. and EDDM (early drift detection method) [33] proposed by Baena et al. have these features. A new base classifier is only created with the last two alerts (warning or drift); with the “possibly change” alert, the new base classifier is associated with the current concept and, with the “change” alert, it is associated with a new concept. If the alert is “no change,” the ensemble remains unchanged.

The drift detector used was DDM. This approach detects changes in the probability distribution of examples. The main idea of this method is to monitor the error-rate produced by a classifier. Statistical theory states that error decreases if the distribution is stable. When the error increases, it means that the distribution has changed [32].

This procedure ensures that new classifiers are only added when necessary; thus, within the ensemble, old concepts remain longer, which allows for a better treatment of recurring concepts (see Algorithm 5).

### 3.4. Deleting a Base Classifier

The algorithm deletes a base classifier when a new classifier is to be added and the maximum limit of classifiers to store has been reached. The removal process takes into account the following aspects: status of classifiers (active-inactive), age and weight of the classifiers, and the number of classifiers associated with a concept. It is always about first deleting an inactive classifier; in its absence (all classifiers are active), it proceeds to remove an active classifier. To avoid a cumbersome pseudocode, explanations are included (see Algorithm 6).

*Option 1.* The oldest inactive classifier that belongs to a concept which has more than one base classifier associated with it is deleted. If all existing concepts are associated with a single base classifier, the oldest inactive classifier is removed, and, with it, the concept itself is also deleted.

*Option 2.* Active classifier with less weight is removed.

When a base classifier is deleted from the ensemble, all of its associated values (weight, status, and concept) are also deleted.

It is always about deleting the oldest classifier but taking into account the highest number of concepts remaining represented within the ensemble. Often the oldest classifier is not deleted, in order to keep a classifier, which is the only representative of a concept within ensemble.

One purpose of this procedure of deleting base classifiers is to maintain representation of all the concepts analyzed, within the ensemble, for as long as possible. This approach favors the treatment of recurring concepts and maintains a high diversity of concepts within the ensemble.

To identify the best configuration with which to test the ensemble, different values of the following parameters were used: *β*_1_, *β*_2_, *θ*, ne, nt, and max.

To identify the best parameter set, approximately 500 different configurations were tested for these parameters. The best and more robust configuration found was *β*_1_ = 0.5; *β*_2  _ = 0.5; ne = 500; nt = 50, and max⁡ = 15.

### 3.5. Analyzing Spatial and Temporal Complexity

In the context of machine learning, it is important to analyze the time and space complexity of the algorithms. This study is even more necessary when the learning process is done from data streams, online, with the possibility of having nonending datasets.

At this point, now the FAE algorithm has been described and we present a detailed analysis of this complexity. We must note that the algorithm can be configured with different base classifiers, so the final details about complexity will depend on the final base classifier used. In this case, the common configuration that we propose uses the Hoeffding Tree or VFDT [34] and the analysis of complexity is done under this assumption (additional studies for different base classifiers can be easily derived).

*Spatial complexity* is basically determined by the maximum number of base classifiers stored in the ensemble (max) and their maximum size. In our case, we have used decision trees, so the maximum size for such a model is a completely expanded decision tree. If we assume that the dataset is defined by a finite number of attributes (*n*_attr) with a maximum number of symbolic values (*n*_values), the spatial complexity is *O*(max⁡·*n*_attr^*n*_values^), which is polynomial. This reasoning is extensible to numerical attributes. In worst case, there will be as many different values as different examples in the set used to build the base classifier (ne). In addition, it is easy to have fewer values because discretization methods can be applied [35]. Clearly, this is the worst case. In general, the amount of space needed is much lower.

*Temporal complexity*, in this context, cannot be studied for the whole process, because it is continuous. It is usual to study the processing time per example or, in our case, per block of examples (ne or nt). Therefore, the analysis can be done according to two situations: building new base classifiers or updating them. In the first case, the temporal complexity depends on the temporal complexity for the selected base classifier. In our case, we have configured FAE to use VFDT, so it requires constant time to process each example [34] (which will depend on the size of a completely expanded tree), that is, *O*(ne · *n*_attr^*n*_values^). In the second case, the updating process, each example in the testing block (with size nb) is tested with each base classifier in order to update all the weights and statuses. In the worst case scenario, the classifiers are completely expanded decision trees (whose branches have as nodes as attributes, *n*_attr), so the temporal complexity is *O*(nb · max⁡·*n*_attr).

## 4. Experimental Results

In this section, we present the algorithms used in the tests, parameters, information about the datasets, and an empirical study of the results obtained.

The proposal presented here was implemented using the Massive Online Analysis (MOA) framework [36], developed at Waikato University, New Zealand. MOA is a framework for mining data streams. It offers a collection of machine learning algorithms, evaluation tools, and dataset generators commonly used in data stream research.

Experiments were performed on a computer using an Intel, Pentium CPU, P6000 1.87 GHz with a RAM of 4 GB.

*Algorithms.* In the experiments, we used the following algorithms: AWE (Wang et al., 2003), AUE (Brzezinski and Stefanowski, 2011), OzaBagAdwin (Oza and Russell, 2009), and decision trees with Hoeffding bounds or Very Fast Decision Tree (Hoeffding Tree or VFDT, Domingos and Hulten, 2000); all the algorithms have freely available implementations in the MOA framework.

The base classifier used in the experiments for all algorithms (ensembles) was a Hoeffding Tree or VFDT [34]. So we can exclude the influence of the base classifier in the comparison between ensembles. It is also important to compare the Hoeffding Tree with the rest of the algorithms to verify whether they improve it or not.

The parameters used for each of the algorithms (AWE, AUE, OzaBagAdwin, and Hoeffding Tree) in the experiments are the default values defined in the MOA framework.

*Artificial Datasets.* We selected two artificial datasets to perform the experiments: LED, proposed by Breiman et al. in 1984 and SEA proposed by Nick Street and Kim in 2001. They are commonly used in the concept drift research area [1] and are freely available from the MOA framework.

The LED dataset is composed of 24 categorical attributes; 17 of which are irrelevant, and one categorical class with ten possible values. The goal is to predict the digit displayed on a seven-segment LED display, where each attribute has 10% probability of being inverted (noise) [36]. We used a version of LED available at MOA that includes concept drifts in the datasets by simply changing the attribute positions.

The SEA dataset is generated using three attributes, where only the first two are relevant. All three attributes have values between 0 and 10. The points of the dataset are divided into 4 different concepts. The classification is done using *f*1 + *f*2 ≤ *α*, where *f*1 and *f*2 represent the first two attributes and *α* is a threshold value [36]. The most frequent values of  *α* are 9, 8, 7, and 9.5. We also used a version of SEA concept available at MOA.  Table 1 shows general characteristics of LED and SEA datasets.

To create abrupt or gradual concept drifts in data stream, the MOA framework uses a sigmoid function, as a practical solution for defining the probability that each new example of the stream will belong to the new concept after the drift. In this sigmoid model, only two parameters need to be specified: *t*_0_, the point of change, and *w*, the length of change [36] (number of examples in the transition between concepts).

### 4.1. Abrupt and Gradual Change

In a first phase of the experiments to test the behavior of the algorithms under consideration, over different concept drifts, the following schemas were used: 100000 examples, half of the examples of the first concept and the second concept of a remainder (*t*_0_ = 50000). The length of change (*w*) takes four possible values 0, 100, 500, and 1000 to simulate an abrupt change from (*w* = 0) to more gradual changes (*w* = 1000).

Table 2 shows the results of testing on the LED dataset. The first 50000 examples were generated with a number of drifting attributes equal to 1 and the other 50000 with a number of drifting attributes equal to 7.

Table 2 shows that the accuracy significantly reduced around the change point (transition between concepts). FAE always reported results between the two best accuracy values taken around the change point for all values of *w* (0, 100, 500, and 1000). The same applies to the other two results, final accuracy and time.

**Figure 1:**
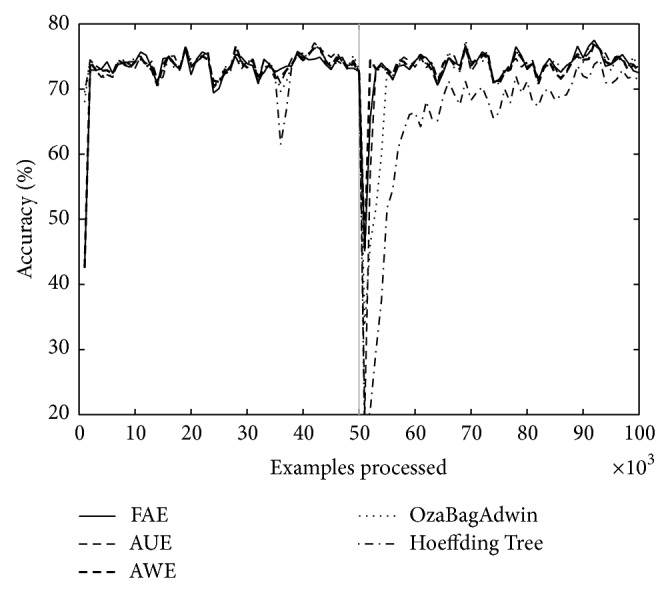
LED concept, 100 000 examples. One change point: *t*_0_ = 50000, *w* = 0.

**Figure 2:**
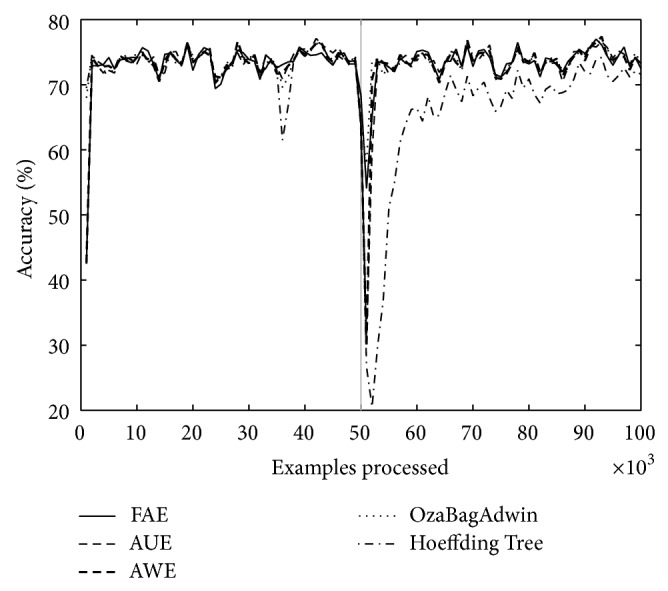
LED concept, 100 000 examples. One change point: *t*_0_ = 50 000, *w* = 1000.

Figures 1 and 2 correspond to the results of Table 2 (*w* = 0, columns A and *w* = 1000, columns D). We can see that the graphs show accuracy falls around the change point. We consider it important to draw attention to the depth and width of each of these falls; depth indicates by how much accuracy falls for each algorithm around the change point and the width indicates how long it takes to recover. FAE reports results with a low loss of accuracy both in plotted results and in the rest of the experiments (not plotted) and a low recovery time compared to the other algorithms.

Very similar results to those described above occur when testing on data generated according to SEA concept. The first 50000 examples were generated with the first classification function (*f*1 + *f*2 ≤ 9) and the others with the fourth classification function (*f*1 + *f*2 ≤ 9.5) (see Table 3).

Over both datasets, LED and SEA, and over different types of changes, abrupt and gradual ones, FAE shows promising results with regard to accuracy fall depth around the change point, recover time from accuracy fall (see Figures 1 and 2), final accuracy, and runtime.

### 4.2. Recurring Concept Drift

In the second phase of the experiments to test the behavior of the algorithms over recurring concepts, we built 8 datasets, each one with 100000 examples and in the presence of recurring concepts. 

*Dataset 1 and 2.* LED concept, three change points, every 25000 examples, we change the number of attributes with drift (we follow the scheme 1, 7, 1, 7; number of attributes with drift). Dataset 1, *w* = 0, and dataset 2, *w* = 1000.

*Dataset 3 and 4.* SEA concept, three change points, every 25000 examples, we change thethreshold value *α* (we follow the scheme 9.5, 9, 9.5, 9 threshold value). Dataset 3, *w* = 0, and dataset 4, *w* = 1000.

*Dataset 5 and 6.* LED concept, seven change points, every 12500 examples, we change the number of attributes with drift (we follow the scheme 1, 3, 5, 7, 1, 3, 5, 7; number of attributes with drift). Dataset 5, *w* = 0, and dataset 6, *w* = 1000.

*Dataset 7 and 8.* SEA concept, seven change points, every 12500 examples, we change the threshold value *α* (we follow the scheme 7, 8, 9.5, 9, 7, 8, 9.5, 9 threshold value). Dataset 7, *w* = 0, and dataset 8, *w* = 1000.

**Figure 3:**
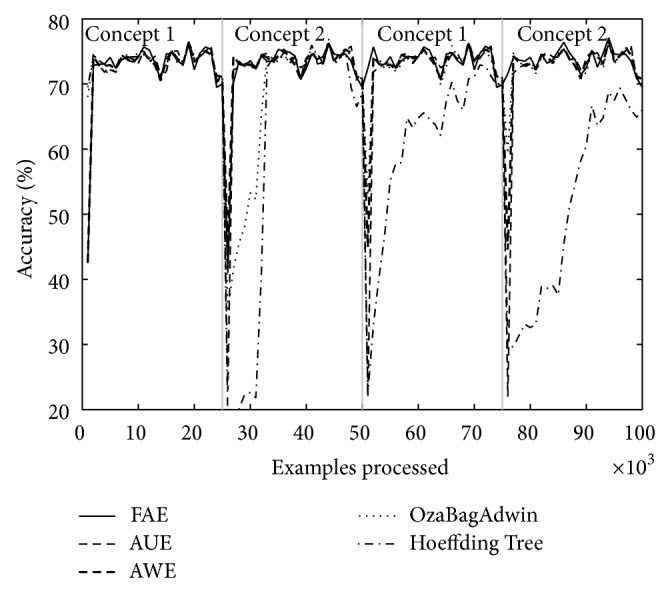
Dataset 1. LED concept, three change points: every 25 000 examples, *w* = 0.

**Figure 4:**
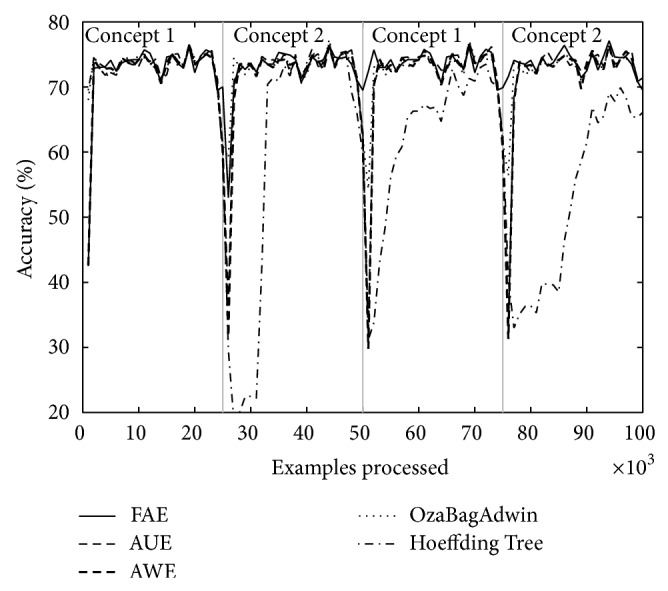
Dataset 2. LED concept, three change points: every 25 000 examples, *w* = 1000.

**Figure 5:**
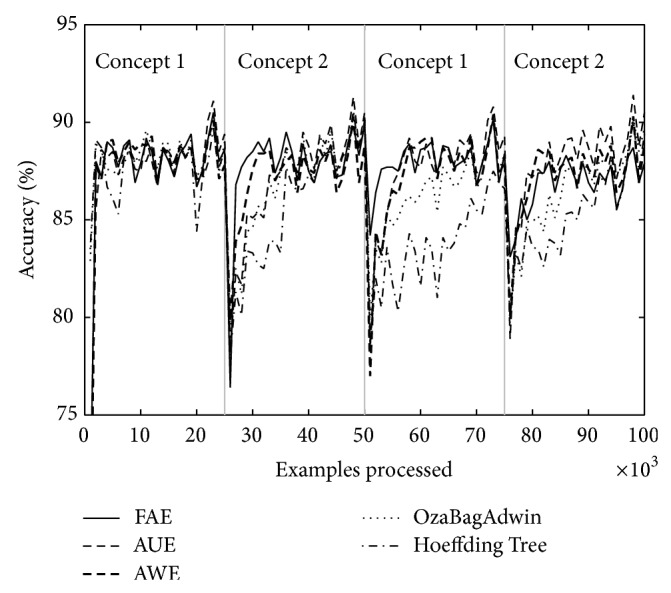
Dataset 3. SEA concept, three change points: every 25 000 examples, *w* = 0.

**Figure 6:**
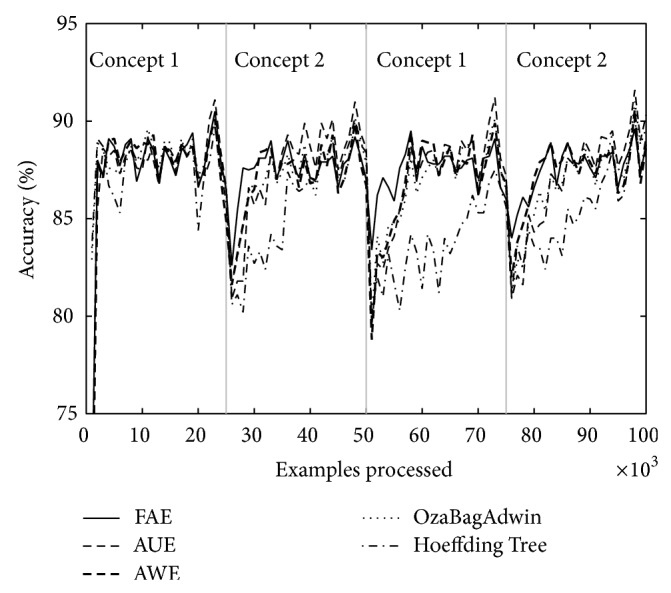
Dataset 4. SEA concept, three change points: every 25 000 examples, *w* = 1000.

**Figure 7:**
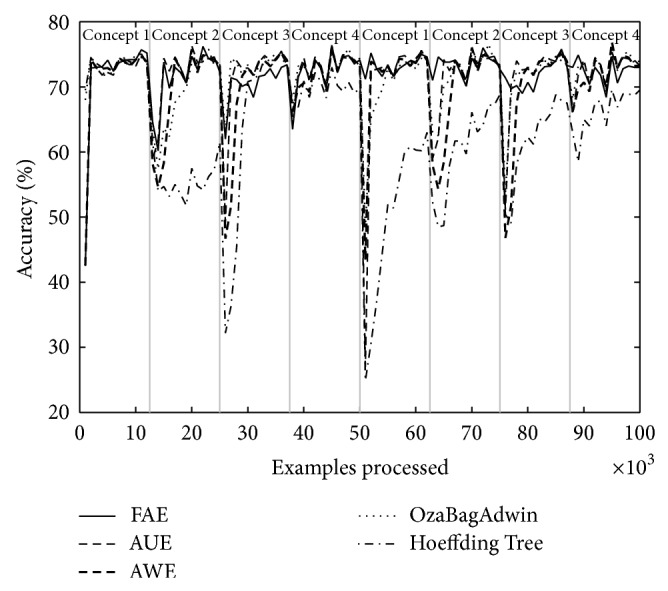
Dataset 5. LED concept, seven change points: every 12 500 examples, *w* = 0.

**Figure 8:**
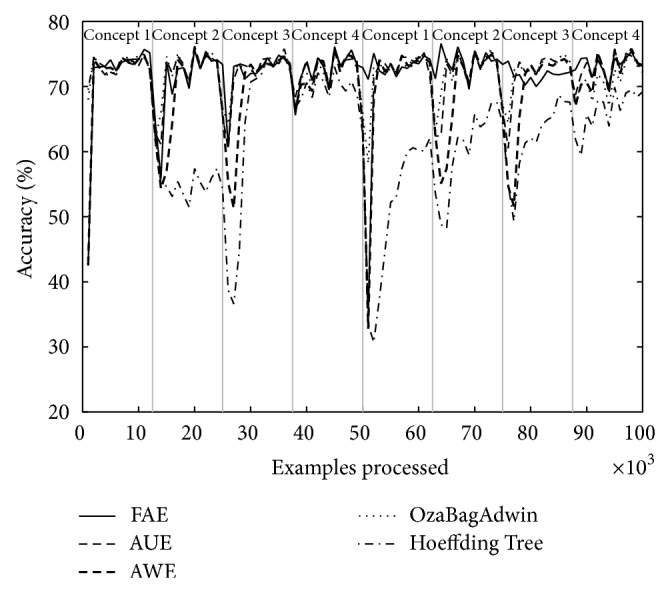
Dataset 6. LED concept, seven change points: every 12 500 examples, *w* = 1000.

**Figure 9:**
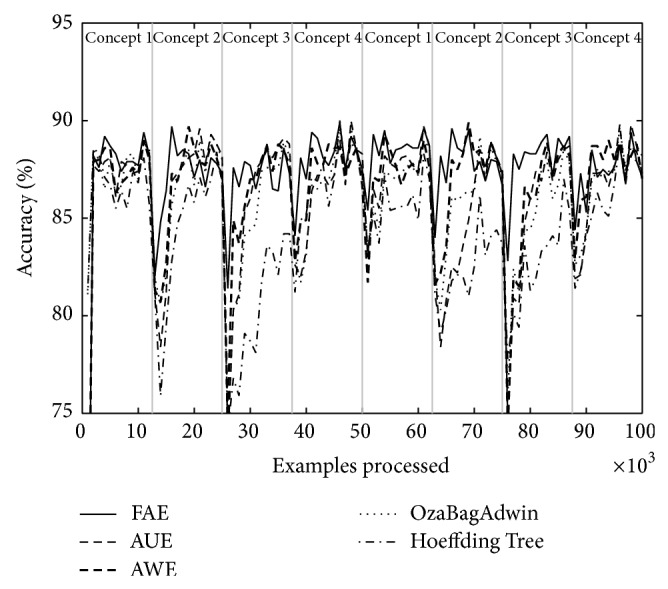
Dataset 7. SEA concept, seven change points: every 12 500 examples, *w* = 0.

**Figure 10:**
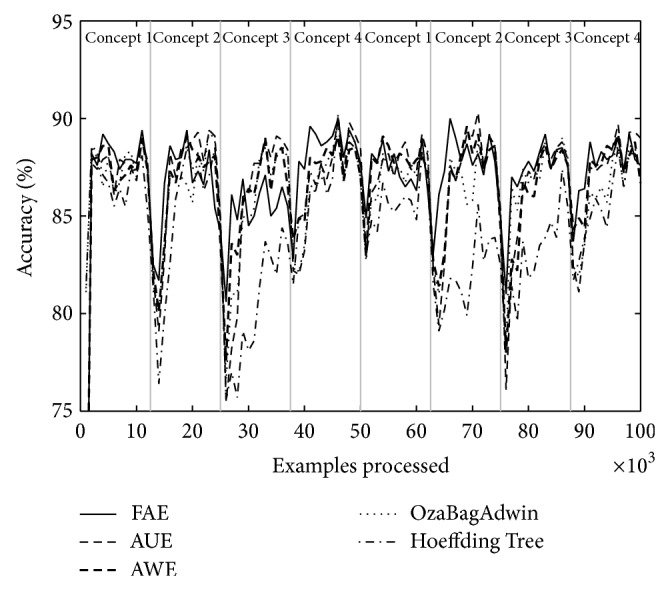
Dataset 8. SEA concept, seven change points: every 12 500 examples, *w* = 1000.

Tables 4 and 5 show the results of evaluating each of the algorithms over the eight datasets defined above.  Table 4 shows the results when there are four concepts and Table 5 when there are eight. Each table comes with four figures (Table 4 with Figures 3, 4, 5, and 6; Table 5 with Figures 7, 8, 9, and 10) which plot accuracy values as functions of processed examples.

Labels, concept 1, concept 2…, were added only to the figures in order to visually highlight when a concept is recurrent. The same concept is labeled with the same label.

According to the results shown in Tables 4 and 5, it is interesting to note the following.

The Hoeffding Tree algorithm (it is not an ensemble) always achieves better results than other algorithms in terms of runtime; however, it gets the worst final accuracy values in all cases. By contrast, the algorithm AWE has the worst results regarding runtime in all cases; however, its final accuracy values are comparably good and are included among the best two results in several experiments. The algorithms AUE and OzaBagAdwin achieve comparably good final accuracy values too. The algorithm OzaBagAdwin achieves values very similar to those of FAE algorithm in terms of runtime, although with lower results.

As seen in Tables 4 and 5, FAE always reported results between the two best accuracy and runtime values. These values show that the new approach achieves better results than the rest of the algorithms over the datasets with the proposed features (concept drifts and recurring concepts).

In each figure, we consider it important to draw attention to the second half starting from the 50000th example, when previously analyzed concepts reappear (recurring concepts). We can see that FAE shows practically no falls in the accuracy values compared to the rest of the algorithms. Differences are more notable over the LED dataset. FAE is an algorithm built to deal with recurring concepts and this is precisely what the results show. According to the results of the experiments, FAE is able to handle abrupt and gradual concept drifts; moreover, it is far superior to the rest of the algorithms in the treatment of recurring concepts.

### 4.3. Real Dataset

The real world dataset we work with in this section has been used in several studies about concept drift [37]. For this dataset, there is no strong claim about any presence or type of change. In this dataset, we evaluate the algorithms by processing the examples in their temporal order.

Electricity dataset (Elec2) was described by Harries and analyzed by Gama. This dataset was collected from the Australian New South Wales Electricity Market. In this market, prices are not fixed and are affected by demand and supply of the market. They are set every five minutes. The Elec2 dataset contains 45, 312 examples. The class label identifies the change of the price relative to a moving average of the last 24 hours.

**Figure 11:**
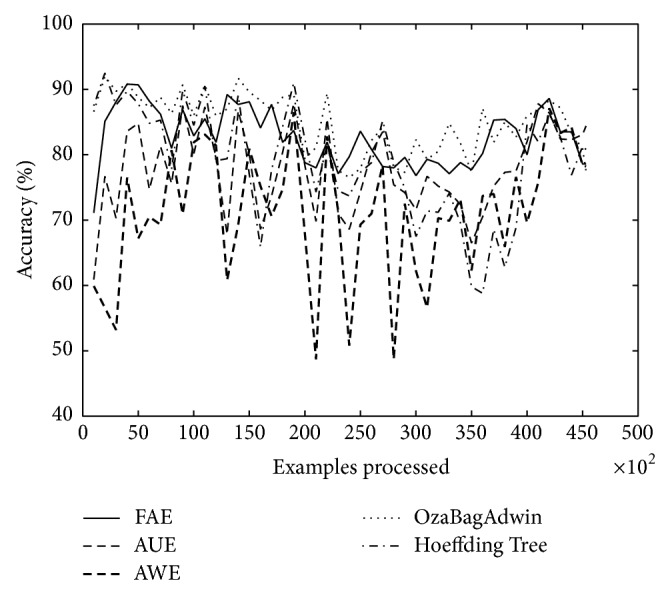
Electricity dataset, 45 312 examples.

As seen in Table 6 and in Figure 11, FAE reported results between the two best accuracy values again. However, it is important to note that the OzaBagAdwin algorithm achieved the best results over Electricity dataset.

## 5. Conclusions

The treatment of large data streams in the presence of concept drifts is one of the main challenges in the data mining area, specifically when the algorithms have to deal with concepts that disappear and then reappear. Most algorithms concentrate their efforts on the current data, by deleting or modifying previously constructed models that represent concepts that have disappeared. Many times when these concepts reappear, algorithms have to repeat work already done. This paper has presented FAE, an algorithm that adapts very quickly to both abrupt and gradual concept drifts, and has been specifically built to deal with recurring concept drifts.

FAE stores a set of inactive base classifiers (while, in this status, they are not used for prediction) which represent old concepts that were analyzed and then disappeared. These classifiers change to active status very quickly when the concept that they represent reappears.

FAE uses a drift detector (often DDM is used) to decide when to build and add a new base classifier. This mechanism allows adding new base classifiers only when necessary, thus contributing to saving memory which is used to keep other models. Using a drift detector favors the treatment of abrupt concept drifts, which is combined with the natural treatment of ensembles for gradual concept drifts.

FAE uses a weighted majority vote to obtain the global ensemble decision and proposes a formula for adjusting the weights of the base classifiers that allows the algorithm to increase or decrease them in relation to their actual performance. This mechanism allows the base classifiers to remain longer within the ensemble.

The experiments carried out show promising results of the proposed algorithm over datasets generated according to LED and SEA concepts and the real world dataset. Both abrupt and gradual concept drifts as well as the existence of recurring concepts have been simulated.

## Figures and Tables

**Algorithm 1:**
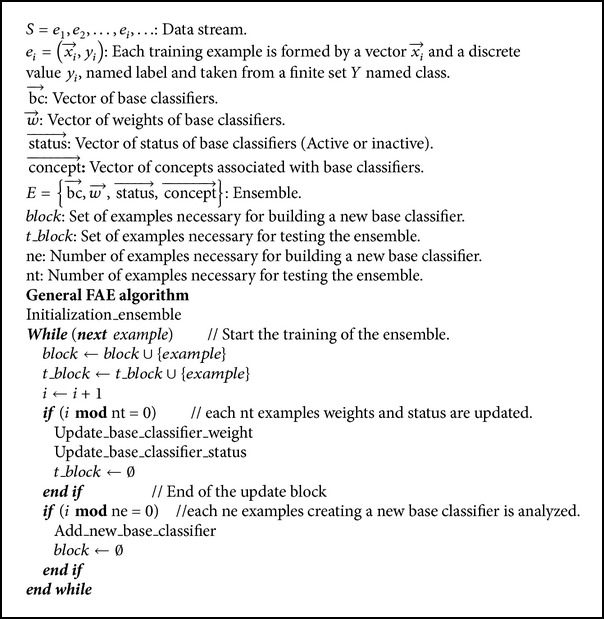


**Algorithm 2:**
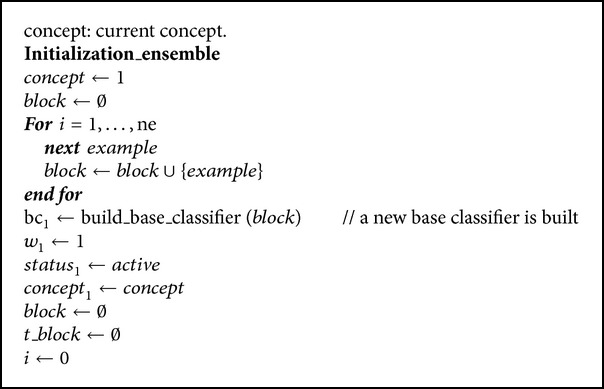


**Algorithm 3:**
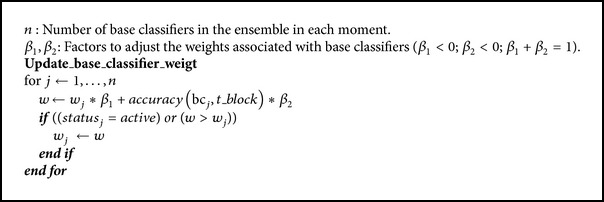


**Algorithm 4:**
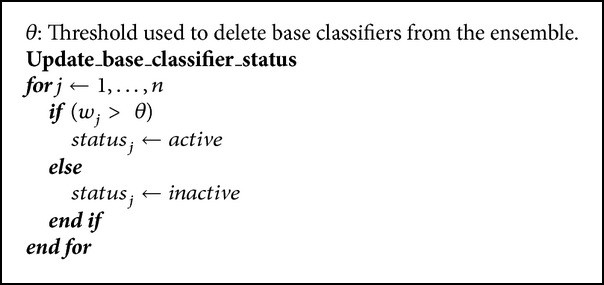


**Algorithm 5:**
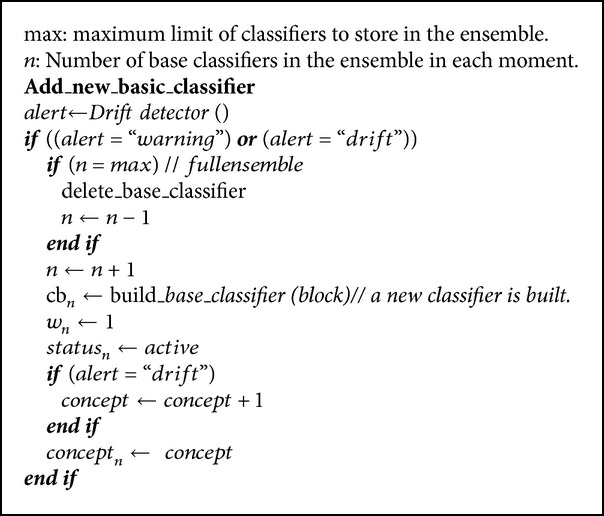


**Algorithm 6:**
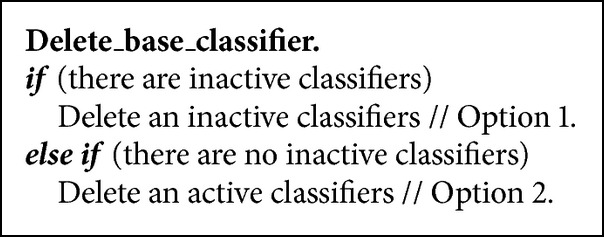


**Table 1:** General characteristics of LED and SEA datasets.

Dataset	Attribute number	Relevant attribute	Irrelevant attribute	Total sample number
LED	24	7	17	100 000
SEA	3	2	1	100 000

**Table 2:** LED concept, 100 000 examples. One change point: *t*_0_ = 50 000. Columns A, *w* = 0; columns B, *w* = 100; columns C, *w* = 500; and columns D, *w* = 1000.

Classifiers	Lowest accuracy around change point (%)				Final accuracy (%)				Time (s)			
	A	B	C	D	A	B	C	D	A	B	C	D
FAE	**45,1**	**50,9**	**55,1**	**54,1**	**73,15**	**73,2**	**73,16**	**73,15**	**63,01**	**64,55**	**66,75**	**68,09**
AUE	19,9	15,6	26,6	31	72,84	72,99	72,95	72,89	128,12	118,81	109,25	125,49
AWE	**45,2**	44,7	27,5	29,7	**73,13**	73,12	72,97	72,87	234,52	238,35	230,43	204,13
OzaBagAdwin	33,6	**61,6**	**61,1**	**57,8**	72,68	**73,53**	**73,48**	**73,44**	101,57	102,13	101,43	102,68
Hoeffding Tree	16,9	18	21,7	26,7	69,24	69,24	69,26	69,24	**9,05**	**8,95**	**8,36**	**9,05**

**Table 3:** SEA concept, 100 000 examples. One change point: *t*_0_ = 50 000. Columns A, *w* = 0; columns B, *w* = 100; columns C, *w* = 500; and columns D, *w* = 1000.

Classifiers	Lowest accuracy around change point (%)				Final accuracy (%)				Time (s)			
	A	B	C	D	A	B	C	D	A	B	C	D
FAE	78,3	**79,5**	**79,6**	**80,5**	**88,1**	88,14	**88,14**	88,07	**8,19**	**8,17**	**8,03**	**8,05**
AUE	78,2	78,6	79,3	**79,5**	**88,11**	**88,15**	**88,32**	**88,09**	15,33	14,76	14,57	15,46
AWE	**80,7**	**80,8**	**81,7**	82	87,69	**87,68**	87,73	87,72	31,04	29,89	26,64	27,85
OzaBagAdwin	**78,5**	78,8	79,2	79,2	87,99	88,07	87,83	**88,25**	12,73	13,68	13,88	10,9
Hoeffding Tree	78,1	78,5	79	79,1	86,81	86,8	86,8	86,8	**1,08**	**1,12**	**1,09**	**1,09**

**Table 4:** LED and SEA concepts, 100 000 examples. Three change points: every 25 000 examples. Columns A, *w* = 0 and columns B, *w* = 1000.

Classifiers	LED				SEA			
	Final accuracy (%)		Time (s)		Final accuracy (%)		Time (s)	
	A	B	A	B	A	B	A	B
FAE	**72,65**	**72,36**	**83,9**	**82,98**	**87,52**	**87,48**	**14,6**	**11,72**
AUE	71,82	71,79	142,23	143,58	**87,51**	**87,23**	15,12	15,23
AWE	**72,43**	71,52	233,14	239,77	87,3	87,17	31	30,84
OzaBagAdwin	71,39	**72,6**	99,23	84,26	86,97	87,21	13,29	13,42
Hoeffding Tree	61,22	61,79	**8,07**	**5,32**	85,61	85,6	**1,05**	**1,15**

**Table 5:** LED dataset, 100 000 examples. Seven change points: every 12 500 examples. Columns A, *w* = 0 and columns B, *w* = 1000.

Classifiers	LED				SEA			
	Final accuracy (%)		Time (s)		Final accuracy (%)		Time (s)	
	A	B	A	B	A	B	A	B
FAE	**72,27**	**71,95**	**74,69**	**81,32**	**87,46**	**86,95**	**11,37**	**10,67**
AUE	71,52	71,59	133,54	120,84	85,99	86,4	15,09	14,96
AWE	70,38	70,06	236,58	210,79	**86,58**	**86,5**	30,62	30,17
OzaBagAdwin	**71,68**	**72,08**	86,67	91,37	86,13	86,31	12,99	13,23
Hoeffding Tree	62,65	62,71	**8,5**	**7,92**	84,31	84,35	**1,09**	**1,19**

**Table 6:** Electricity dataset, 45 312 examples.

Electricity (elec2)		
Classifiers	Final accuracy (%)	Time (s)
FAE	**82,4**	9,34
AUE	77,7	10,62
AWE	71,13	12,89
OzaBagAdwin	**84,81**	**7,77**
Hoeffding Tree	78,92	**1,53**

## References

[1] Gama J., Zliobaite I., Bifet A., Pechenizkiy M., Bouchachia A. (2014). A survey on concept drift adaptation. *ACM Computing Surveys*.

[2] Widmer G., Kubat M. (1996). Learning in the presence of concept drift and hidden contexts. *Machine Learning*.

[3] Ferrer-Troyano F. J., Aguilar-Ruiz J. S., Riquelme J. C. (2005). Incremental rule learning and border examples selection from numerical data streams. *Journal of Universal Computer Science*.

[4] Minku L. L., White A. P., Yao X. (2010). The impact of diversity on online ensemble learning in the presence of concept drift. *IEEE Transactions on Knowledge and Data Engineering*.

[5] Tsymbal A. (2004). The problem of concept drift: definitions and related work.

[6] del Campo-Ávila J. (2007). *Nuevos Enfoques en Aprendizaje Incremental [Ph.D. thesis]*.

[7] Gama J., Medas P., Castillo G., Rodríguez P. Learning with drift detection.

[8] Nick Street W., Kim Y. A streaming ensemble algorithm (SEA) for large-scale classification.

[9] Kolter J., Maloof M. Dynamic weighted majority: a new ensemble method for tracking concept drift.

[10] Wang H., Fan W., Yu P. S., Han J. Mining concept-drifting data streams using ensemble classifiers.

[11] Deckert M. (2011). *Batch Weighted Ensemble for Mining Data Streams with Concept Drift*.

[12] Gonçalves P., Barros R. (2013). *RCD: A Recurring Concept Drift Framework*.

[13] Brzezinski D., Stefanowski J., Corchado E., Kurzynski M., Wozniak M. (2011). Accuracy updated ensemble for data streams with concept drift. *Hybrid Artificial Intelligent Systems*.

[14] Littlestone N., Warmuth M. K. (1994). The weighted majority algorithm. *Information and Computation*.

[15] Kolter J. Z., Maloof M. A. Using additive expert ensembles to cope with concept drift.

[16] Yue S., Guojun M., Xu L., Chunnian L. Mining concept drifts from data streams based on multiclassifiers.

[17] Mejri D., Khanchel R., Limam M. (2013). An ensemble method for concept drift in nonstationary environment. *Journal of Statistical Computation and Simulation*.

[18] Blum A. (1997). Empirical support for winnow and weighted-majority algorithms: results on a calendar scheduling domain. *Machine Learning*.

[19] Minku L. L., Yao X. (2012). DDD: a new ensemble approach for dealing with concept drift. *IEEE Transactions on Knowledge and Data Engineering*.

[20] Breiman L. (1996). Bagging predictors. *Machine Learning*.

[21] Freund Y. Boosting a weak learning algorithm by majority.

[22] Oza N., Russell S. (2001). Online bagging and boosting. *Artificial Intelligence and Statistics 2001*.

[23] Bifet A., Holmes G., Pfahringer B., Kirkby R., Gavaldà R. New ensemble methods for evolving data streams.

[24] Bifet A., Gavaldà R. Learning from time-changing data with adaptive windowing.

[25] Wankhade K. K., Dongre S. S. (2012). A new adaptive ensemble boosting classifier for concept drifting stream data. *International Journal of Modeling and Optimization*.

[26] Dongre S., Malik L. (2013). Algorithm to handle concept drifting in data stream mining. *IJCSN International Journal of Computer Science and Network*.

[27] Nishida K., Yamauchi K., Omori T. (2005). ACE: adaptive classifiers-ensemble system for concept-drifting environments. *Multiple Classifier Systems*.

[28] Ramamurthy S., Bhatnagar R. Tracking recurrent concept drift in streaming data using ensemble classifiers.

[29] Li P., Wu X., Hu X. (2010). Mining recurring concept drifts with limited labeled streaming data. *Journal of Machine Learning Research—Proceedings Track*.

[30] Gama J., Kosina P. (2009). *Tracking Recurring Concepts with Meta-Learners*.

[31] Tanenbaum A. (1988). *Computer Networks*.

[32] Gama J., Medas P., Castillo G., Rodrigues P. Learning with drift detection.

[33] Baena M., del Campo J., Fidalgo R., Bifet A., Gavaldá R., Bueno R. M. Early drift detection method.

[34] Domingos P., Hulten G. Mining high-speed data streams.

[35] Mora L. l., Fortes I., Morales-Bueno R., Triguero F. (2000). Dynamic discretization of continuous values from time series. *Book “ECML'00”*.

[36] Bifet A., Holmes G., Kirkby R., Pfahringer B. (2010). MOA: massive online analysis. *Journal of Machine Learning Research*.

[37] Bártolo J. (2011). *Learning recurring concepts from data stream in ubiquitous environments [Ph.D. thesis]*.

